# Book Notices

**Published:** 1883-06-20

**Authors:** 


### *BOOK NOTICES.*

A TREATISE ON THERAPEUTICS, Comprising Materia Medica and Toxicology, with Especial reference to the Application of the Physiological action of Drugs to Clinical Medicine, by H. C. Wood, M. D., Professor of Materia Medica and Therapeutics, and Clinical Professor of Diseases of the Nervous System, in the University of Pennsylvania; Physician to the Philadelphia Hospital; Member of the National Academy of Science, etc. Fifth Edition, revised and enlarged. Philadelphia: J. B. Lippincott & Co., 1883.

This work contains 740 octavo pages and is ably written. The author states that as a teacher of Therapeutics, the work grew out of a need felt by himself, and his apology for addressing another Treatise to this department, and his reasoning upon the lessons to be derived from the experiments upon the lower animals as found in his preface, are very clear and satisfactory. The need, he remarks, is "for a book into which should be gathered the many scattered facts in regard to the physiological action of medicine—a book in which an attempt should be made to sift the true from the false, to reconcile seeming differences, to point out what we know and what we do not know; and, to give a platform from which investigators might start forward without the necessity of being, as is so often the case, ignorant of what was already achieved, or of spending a great deal of time in a wild hunt through the almost boundless, but often scattered and inaccessible ranges of Continental literature.

The fourth edition of the work was quickly exhausted, and now the fifth edition is presented, representing, it is believed, more thoroughly than ever before, the best therapeutic thoughts of the day.

THE MICROSCOPE AND ITS REVELATIONS. By William B. Carpenter, C. B., M. D., LL.D., F. R. S., F. G. S., F. L. S., Corresponding member of the Institute of France and of the American Philosophical Society, etc., etc. Sixth edition, illustrated by twenty-six plates and five hundred wood engravings. Vol. II. New York: William Wood & Co.

A work of 354 octavo pages, containing much matter of exceeding interest and novelty in reference to microscopic forms of animal life, embracing Foramenifora and Rodiolara, Sponges and Zoophytes, Polyzoa and Tunicata, Molluscous animals, Insects and Arachinda. The microscope in Geological investigations, Crystallization, Polarization, etc. This is one of Wood's Library of Standard Medical Authors, and must prove very interesting and instructive to the Medical and especially to the Scientific reader.

THE PATHOLOGY AND TREATMENT OF DISEASES OF THE OVARIES, being the Hastings Essay for 1873. By Lawson Tait, F. R. C. S., Edin. and Eng., Surgeon to the Birmingham Hospital for Women, and Consulting Surgeon to the West Bromwich Hospital. Fellow of the Royal Medico-Chirurgical Society; member of the Surgical Society of Ireland, and of the Medico-Chirurgical Society of Edinburg, Etc., Etc. Fourth edition, re-written and greatly enlarged. New York: William Wood & Co., 56 LaFayette Place, 1883.

The work is practical in character, giving the anatomy and physiology of the Ovaries and the various diseases pertaining thereto. Ovariotomy, with the recent extensions of abdominal and pelvic surgery. It is exceedingly interesting and instructive in the department of which it treats. Illustrated. Neatly printed. Cloth, 357 octavo pages.

TRANSACTIONS OF THE MEDICAL SOCIETY OF THE STATE OF GEORGIA, for the thirty-third Annual Session, held in Atlanta, April 16th, 1882.

The book is neatly gotten up in cloth, and numbers 226 octavo pages. While the work contains a number of interesting papers, it is yet a small volume of Transactions for the Empire State of the South. The Secretary's Report details no small amount of labor and trouble on his part, to get out the book, as the assessments against the members are but partially paid and the cost of printing very considerable. The interest in the Society does not seem sufficient to bring out a full attendance of its membership. It is hoped that the next meeting, which is appointed for Macon, Georgia, on the first Wednesday in April, 1884, will be more largely attended, and that measures will be devised to bring about an increased interest in the Association.

INTERNATIONAL ENCYCLOPÆDIA OF SURGERY, A Systematic Treatise on the Theory and Practice of Surgery, by authors of various nations. Edited by John Ashhurst, Jr., M. D., Prof. of Clinical Surgery in the University of Pennsylvania. Illustrated with chromo-lithographs and wood cuts—in six volumes. Vol. III. New York: Wm. Wood & Co., 1883.

The above is volume third of this great work now in process of preparation. It resumes the consideration of Injuries and Surgical diseases of the vascular system.

Among the names of the contributors to the work we note Edmond Andrews, Richard Barwell, Edward Bellamy, P. S. Connor, John A. Liddell, M. Nicaise, John A. Wyeth. Touching reference is made by the author to the death of Prof. W. H. Van Buren, of New York, who was one of the colaborators. As evidence of the high value placed upon the work it may be stated that the first two volumes have been translated into both the French and Italian languages. We have not space to detail the excellencies of the work. Let it suffice to say in general terms that the present volume is fully up to those previously published, and is in all respects an excellent work. The articles of which it treats being considered in an able and exhaustive manner.

GOUT IN ITS PROTEAN ASPECTS. By J. Milner Fothergill, M. D., member of the Royal College of Physicians of London; Physician to the City of London Hospital for Diseases of the Chest; Associate Fellow of the College of Physicians of Philadelphia. Detroit, Michigan: Geo. S. Davis, publisher, 1883; 300 pages.

This work we regard as eminently practical and much needed. In this country most affections of a gouty character are classed as rheumatic, and the majority of practitioners make no distinction, and pay little regard to the physiological causes of the various rheumatic and gouty affections which prevail. Few practitioners, we opine, can read this book without learning something at once instructive, practical and important.

THE DISEASES OF WOMEN, A MANUAL FOR PHYSICIANS AND STUDENTS. By Heinrich Fritsch, M. D, Professor of Gynecology and Obstetrics at the University of Halle. Translated by Isidor Hurst, with 150 wood engravings. New York: Wm. Wood & Co., 1883.

This is an interesting and valuable work of 355 octavo pages, in which we have the department of Gynecology considered under the following general heads: Anatomy and Physiology of the Genital Organs; General Diagnosis; Antisepsis; General Therapeutics; Diseases of the Vulva and Vagina; Diseases of the Bladder and Urethra; Uterine Malformations, etc.; Inflammation of the Uterus, Metritis, etc.; Diseases of Endometrium; Dislocations of Uterus; New Formations; Diseases of the Pelvic Connective Tissue, etc,; Diseases of the Ovaries; Hysteria.

